# Fast Recognition of *Lecanicillium* spp., and Its Virulence Against *Frankliniella occidentalis*

**DOI:** 10.3389/fmicb.2020.561381

**Published:** 2020-10-22

**Authors:** Yeming Zhou, Xiao Zou, Junrui Zhi, Jiqin Xie, Tao Jiang

**Affiliations:** ^1^Institute of Entomology, Guizhou University, The Provincial Key Laboratory for Agricultural Pest Management of Mountainous Region, Guiyang, China; ^2^Institute of Fungus Resources, Guizhou University, Guiyang, China

**Keywords:** entomopathogenic fungi, fast recognition, *Frankliniella occidentalis*, *Lecanicillium*, pathogenicity

## Abstract

**Background:**

*Frankliniella occidentalis* (Thysanoptera: Thripidae) is a highly rasping-sucking pest of numerous crops. The entomogenous fungi of *Lecanicillium* spp. are important pathogens of insect pests, and some species have been developed as commercial biopesticides. To explore *Lecanicillium* spp. resources in the development of more effective *F. occidentalis* controls, efficient barcode combinations for strain identification were screened from internal transcribed spacers (ITS), *SSU*, *LSU*, *TEF*, *RPB1*, and *RPB2* genes.

**Results:**

Six genes were used to reconstruct *Lecanicillium* genus phylogeny. The results showed that ITS, *TEF*, *RPB1*, and *RPB2* could be used to identify the strains. All phylogenetic trees reconstructed by free combination of these four genes exhibited almost the same topology. Bioassay studies of a purified conidial suspension further confirmed the infection of second-instar nymphs and adult female *F. occidentalis* by seven *Lecanicillium* strains. *L. attenuatum* strain GZUIFR-lun1405 was the most virulent, killing approximately 91.67% *F. occidentalis* adults and 76.67% nymphs after a 7-day exposure. *L. attenuatum* strain GZUIFR-lun1405 and *L. cauligalbarum* strain GZUIFR-ZHJ01 were selected to compare the fungal effects on the number of eggs laid by *F. occidentalis*. The number of *F. occidentalis* nymphs significantly decreased when *F. occidentalis* adults were treated with *L. cauligalbarum* strain GZUIFR-ZHJ01.

**Conclusions:**

The combination of ITS and *RPB1* could be used for fast recognition of *Lecanicillium* spp. This is the first report of the pathogenicity of *L. attenuatum*, *L. cauligalbarum*, *L araneogenum*, and *L. aphanocladii* against *F. occidentalis*. Additionally, *L. cauligalbarum* strain GZUIFR-ZHJ01 caused high *F. occidentalis* mortality and inhibited the fecundity of the pest.

## Introduction

With the development of molecular methods, it has become easier for biodiversity researchers to identify species by DNA barcoding than by morphological methods (Verkley et al., 2013; Vu et al., 2019). The success of DNA barcoding has facilitated the characterization of bacterial biodiversity in every nook and cranny on the planet (Mau et al., 2014; Afshinnekoo et al., 2015). Compared to bacteria, fungi are difficult to definitively identify because their genomes contain large amounts of non-coding and repetitive DNA (Mohanta and Bae, 2015). Internal transcribed spacer (ITS) sequences are useful for delineating many fungal species (Irinyi et al., 2015; Vu et al., 2016). Unfortunately, ITS sequences from more fungal species are required in GenBank (Hawksworth and Lücking, 2017), and some of the submitted sequences are of poor quality (Nilsson et al., 2006; Vu et al., 2016). Therefore, multilocus (*TEF*, *RPB1*, *RPB2*, *LSU*, and *SSU*) sequence data has been used to identify the phylogenetic relationship of some fungi, including as the Ophiocordycipitaceae (Sung et al., 2007; Kepler et al., 2017). However, it would be laborious if analysis of six genes was required to identify each newly collected strain. Some scholars suggest that ITS regions and at least one protein encoding gene with sufficient genetic variation, such as *TEF* or *RPB2*, should be sufficient for strain identification instead of using conserved 28S or 18S rDNA sequences (Jeewon and Hyde, 2016). Therefore, identifying the minimum ITS and gene combination required for accurate strain identification will save a lot of time.

*Frankliniella occidentalis* (Pergande) is a highly polyphagous herbivore that is an economically pernicious pest of many crops and results in especially serious losses to the vegetable and flower industries (van Rijn et al., 1995; Reitz et al., 2011). *F. occidentalis* feeds on the tender parts of crops and flowers or oviposits, which reduces crop productivity and quality (Shipp et al., 2000). The pest also facilitates the transmission of plant viruses, including tomato spotted-wilt virus and impatiens necrotic-spot virus (Whitfield et al., 2005; Ogada and Poehling, 2015). *F. occidentalis* was first described in California, United States (Morse and Hoddle, 2006). Facilitated by increasing international agriculture exchanges, *F. occidentalis* subsequently became a major worldwide pest after the first pesticide-resistant strain was discovered in the 1970s (Mouden et al., 2017; Reitz et al., 2020). *F. occidentalis* was first reported as an invasive pest in Beijing in 2003 (Zhang et al., 2003). It then gradually spread to many provinces in China, where it has been a dominant pest of floriculture and vegetable crops (Yang et al., 2015). At present, *F. occidentalis* can be found in more than 10 provinces throughout China (Zhao et al., 2017). Additionally, it has been predicted that *F. occidentalis* could spread to, and successfully overwinter in, more northern areas of China because of climate change (Wu et al., 2017).

The most common *F. occidentalis* control measures are chemical pesticides. However, their efficacy is often poor because *F. occidentalis* tends to reside within the flowers and buds, where it is difficult to reach with spray (Loughner et al., 2005; Demirozer et al., 2012; Mouden et al., 2017). Moreover, varying degrees of resistance are developing due to the overuse of pesticides (Zhao et al., 1995; Bielza et al., 2007; Wang et al., 2011; Gao et al., 2012). Therefore, biological control has received increasing amounts of attention. Representative entomopathogenic fungi for *F. occidentalis*, including *Beauveria bassiana* (Gao et al., 2012; Wu et al., 2015; Lee et al., 2017), *Metarhizium anisopliae* (Ansari et al., 2007; Skinner et al., 2012; Koupi et al., 2015), *Lecanicillium lecanii* (Schreiter et al., 1994; Vestergaard et al., 1995; Gouli et al., 2009), and *Isaria fumosorosea* (Ansari et al., 2008; Kivett et al., 2016), are of particular interest to researchers (Wu et al., 2018). Unfortunately, very little is known about the ability of various *Lecanicillium* species other than typical species of *L. lacani* to control *F. occidentalis*. Therefore, identifying new entomopathogenic fungi, and fast recognition of existing species capable of controlling *F. occidentalis*, are priorities for our research group because of the needs and demands of the majority of crop growers (Wu et al., 2018).

As insect pathogens, *Lecanicillium* spp. are effective biological control agents for numerous plant diseases, insect pests, and plant-parasitic nematodes (Goettel et al., 2008). To date, almost 15 *Lecanicillium* spp.-based commercial preparations have been, or are currently being, developed (Faria and Wraight, 2007). Kepler et al. (2017) concluded that *Lecanicillium* should be incorporated into *Akanthomyces* and formally transferred a number of *Lecanicillium* species, including *L. attenuatum*, *L. muscarium*, and *L. sabanense*. However, *Lecanicillium* were not very compatible with *Akanthomyces*. In addition, in later research, the name *Lecanicillium* was used again, for example *L. testudineum* and *L. restrictum* (Crous et al., 2018), *L. subprimulinum* (Huang et al., 2018), *L. coprophilum* (Su et al., 2019). To better use *Lecanicillium* to control *F. occidentalis*, we use the previous taxon to carry out our work.

Presently, more than 30 *Lecanicillium* species have been formally described and listed in the Fungorum Index^1^. Considering that the information provided by ITS limited, researchers have used different genes to identify new *Lecanicillium* species. This approach successfully identified two new *Lecanicillium* species (*L. araneogenum* and *L. uredinophilum*) using multilocus (*TEF*, *RPB1*, *RPB2*, *LSU*, and *SSU*) sequence data (Park et al., 2016; Chen et al., 2017). Two other new species (*L. sabanense* and *L. cauligalbarum*) were identified based on combined multilocus and ITS sequence phylogenetic analysis (Chiriví-Salomón et al., 2015; Zhou et al., 2018a). Therefore, screening for the most efficient gene combinations for fungal strains identification will have particular significance. These data will facilitate the exploitation of *Lecanicillium* spp. resources in a more time and budget efficient manner.

## Materials and Methods

### Entomopathogenic Fungi

Samples were collected between 2014 and 2018 from forests or tea plantations in Guizhou Province, China. Specimens were preliminarily identified as *Lecanicillium* spp. by their morphology. Some specimens had been identified and some had not (Table 1). All strains were deposited in GZAC, Guizhou University, Guiyang. The details of the strains are listed in Table 1.

**TABLE 1:** Specimen information and host information of the species used in this study.

Voucher information	Species	Host	References
GZUIFR-huhu	*Lecanicillium* sp.	Spider	This work
GZUIFR-lun1403	*Lecanicillium* sp.	Soil	This work
GZUIFR-lun1404	*Lecanicillium attenuatum*	*Empoasca flavescens*	Zhou et al., 2016
GZUIFR-lun1405	*Lecanicillium attenuatum*	*Empoasca flavescens*	Zhou et al., 2016
GZUIFR-lun1505	*Lecanicillium aphanocladii*	Soil	Zhou et al., 2018b
GZUIFR-ZHJ01	*Lecanicillium cauligalbarum*	Stemborer	Zhou et al., 2018a
GZU1032Lea	*Lecanicillium araneogenum*	Spider	Chen et al., 2017

### Insects

*F. occidentalis* were collected from vegetable plants in the Guiyang area of Guizhou Province, China, and were used to establish a laboratory colony. The individuals were reared and maintained on kidney bean pods in the laboratory of the Institute of Entomology, Guizhou University. The thrips were reared in an artificial-climate chest, with a photoperiod of 14 h light, 10 h dark at 25 ± 1°C and relative humidity of 70 ± 5%.

### DNA Extraction, PCR Amplification, and Sequencing

Genomic DNA was extracted as previously described (Zhou et al., 2018a). The ITS, *SSU*, *LSU*, *TEF*, *RPB1*, and *RPB2* regions were amplified using previously described primers (Zhou et al., 2018a).

### Comparison of Combination Identification

DNA sequences used in this study were edited using the LASERGENE software (version 6.0, DNASTAR). Sequences downloaded from GenBank^2^ comprised 29 *Lecanicillium* taxa and one sequence from *Simplicillium lanosoniveum* CBS 704.86, which was used as the outgroup (Huang et al., 2018). A total of 46 sequence submissions were selected (Table 2). Multiple sequence alignments for *TEF*, *RPB1*, and *RPB2* were performed in MAFFT with the default settings (Katoh et al., 2009). Multiple sequence alignments for ITS, *LSU*, and *SSU* were conducted using the MUSCLE algorithm (Edgar, 2004) from MEGA 6 (Tamura et al., 2013). A multiple alignment of the combined partial sequences was assembled with MEGA 6 (Tamura et al., 2013) and SEQUENCEMATRIX 1.7.8 (Vaidya et al., 2011). The command “hompart” in PAUP^∗^ 4.0b10 was used for assessment of concordance amongst the genes and the ITS region (Swofford, 2001).

**TABLE 2:** Specimen information and GenBank accession numbers for strains used in this study.

Species	Voucher information	*LSU*	*SSU*	*TEF*	*RPB1*	*RPB2*	*ITS*
*Lecanicillium acerosum* CBS418.81		KM283786	KM283762	KM283810	KM283832	KM283852	EF641893
*L. antillanum* CBS350.85		AF339536	AF339585	DQ522350	DQ522396	DQ522450	AJ292392
*L. aphanocladii* CBS797.84		KM283787	KM283763	KM283811	KM283833	KM283853	
***L. aphanocladii* GZUIFR**-**lun1505**		**MN944423**	**MN963920**	**MT006072**	**MT006062**	**MT006067**	**MN944448**
*L. aranearum* CBS726.73a		AF339537	AF339586	EF468781	EF468887	EF468934	AJ292464
*L. araneicola* BTCC-F35							AB378506
*L. araneogenum* GZU1031Lea		KX845703	KX845705	KX845697	KX845699	KX845701	
***L. araneogenum* GZU1032Lea**		KX845704	KX845706	KX845698	KX845670	KX845702	
*L. attenuatum* CBS402.78		AF339565	AF339614	EF468782	EF468888	EF468935	AJ292434
***L. attenuatum* GZUIFR**-**lun1404**		**MN944421**	**MN963918**	**MT006070**	**MT006060**	**MT006065**	**KT345700**
***L. attenuatum* GZUIFR**-**lun1405**		**MN944422**	**MN963919**	**MT006071**	**MT006061**	**MT006066**	**MN944447**
*L. attenuatum* KACC42493		KM283780	KM283756	KM283804	KM283826	KM283846	
***L. cauligalbarum* GZUIFR**-**ZHJ01**		MH730663	MH730665	MH730667	MH801920	MH801922	MH801924
*L. cauligalbarum* GZUIFR-ZHJ02		MH730664	MH730666	MH730668	MH801921	MH801923	MH801925
*L. dimorphum* CBS345.37		KM283788	KM283764	KM283812	KM283834	KM283854	
*L. dimorphum* CBS363.86		AF339559	AF339608	EF468784	EF468890		
*L. flavidum* CBS300.70D		KM283789	KM283765	KM283813		KM283855	EF641877
*L. fungicola var. aleophilum* CBS357.80		KM283791	KM283767	KM283815	KM283835	KM283856	NR_111064
*L. fungicola var. fungicola* CBS992.69		KM283792	KM283768	KM283816		KM283857	NR_119653
*L. fusisporum* CBS164.70		KM283793	KM283769	KM283817	KM283836	KM283858	AJ292428
*L. kalimantanense* BTCC-F23							AB360356
*L. lecanii* CBS101247		KM283794	KM283770	DQ522359	KM283837	KM283859	JN049836
*L. lecanii* CBS102067		KM283795	KM283771	KM283818	KM283838	KM283860	
*L. longisporum* CBS102072		KM283796	KM283772	KM283819	KM283839	KM283861	
*L. longisporum* CBS126.27		KM283797	KM283773	KM283820	KM283840	KM283862	
*L. muscarium* CBS143.62		KM283798	KM283774	KM283821	KM283841	KM283863	
*L. nodulosum* IMI 338014R			EF513075				EF513012
*L. pissodis* CBS118231		KM283799	KM283775	KM283822	KM283842	KM283864	
*L. primulinum* JCM 18525		AB712263					AB712266
*L. primulinum* JCM 18526		AB712264					AB712267
*L. psalliotae* CBS532.81		AF339560	AF339609	EF469067	EF469096	EF469112	JN049846
*L. psalliotae* CBS101270		EF469081	EF469128	EF469066	EF469095	EF469113	
*L. restrictum* CCF5252				LT626943			LT548279
*L. sabanense* JCHA5		KC875225	KC633251	KC633266		KC633249	KC633232
*L. saksenae* IMI 179841							AJ292432
***L.* sp. GZUIFR**-**huhu**		**MN944419**	**MN963916**	**MT006068**	**MT006058**	**MT006063**	**MN944445**
***L.* sp. GZUIFR**-**lun1403**		**MN944420**	**MN963917**	**MT006069**	**MT006059**	**MT006064**	**MN944446**
*L. subprimulinum* HKAS99548		MG585315	MG585316	MG585317			MG585314
*L. subprimulinum* HKAS99549		MG585319	MG585320	MG585321			MG585318
*L. testudineum* UBOCC-A112180				LT992868			LT992874
*L. testudineum* UBOCC-A116026				LT992867			LT992871
*L. tenuipes* CBS309.85		KM283802	KM283778	DQ522341	KM283844	KM283866	JN036556
*L. uredinophilum* KACC44082		KM283782	KM283758	KM283806	KM283828	KM283848	
*L. uredinophilum* KACC47756		KM283783	KM283759	KM283807	KM283829	KM283849	
*L. wallacei* CBS101237		AY184967	AY184978	EF469073	EF469102	EF469119	EF641891
*Simplicillium lanosoniveum* CBS 704.86		AF339553	AF339602	DQ522358	DQ522406	DQ522464	AJ292396

*The bolded values/terms means the representation is provided by us.*

Bayesian inference (BI) was performed using MRBAYES 3.2 (Ronquist et al., 2012) and maximum likelihood (ML) analysis was performed using RAxML (Alexandros, 2014). The ML and BI analyses were performed for each of the six genes (ITS, *LSU*, *SSU*, *TEF*, *RPB1*, and *RPB2*). Portfolio analysis was performed for ML and BI analyses on the basis of single gene analysis. Nucleotide substitution models were determined by MrModeltest 2.3 (Nylander et al., 2004). For BI, 10 000 000 generations were performed, with one tree selected every 500th generation. For ML, the GTRGAMMA model was used, and a bootstrap analysis with 1,000 replicates was performed to assess statistical support for the tree topology. Phylogenetic trees were viewed with TREEGRAPH^3^.

### Bioassay

The virulence of *Lecanicillium* spp. strains against second-instar nymphs and adult females was tested. Second, the biometrics of the adults and the number of nymphs hatched under following treatment with each strain treatment were compared. Strains that significantly reduced the number of nymphs underwent preliminary exploration of thrip fecundity to assess whether the strain directly affected fertility and hatchability.

### Preparation of Spore Suspension

The strains were routinely grown on PDA. Plates were incubated at 25°C for 14-day and aerial conidia were harvested by flooding the plate with 0.025% Tween-80. The conidial suspension was vortexed for 1 min and filtered through double layers of lens paper. The final spore concentrations were resuspended to 5 × 10^8^ conidia ml^–1^ as determined by counting using a hemocytometer (Shanghai Qiujing Biochemical Reagent Instrument Co., Ltd.).

### Inoculation of Adult Females

Females were inoculated using the leaf dipping method with some modifications (bean pods were used instead of leaves) (Zhang et al., 2008). The bean pods were cut longitudinally into long strips of equal size. They were placed in the prepared spore suspension for 15 s, then removed and allowed to dry naturally. The pods were fixed with a paper clip placed in the fingertip tube. Twenty female adults (2–3-day old) *F. occidentalis* were transferred with suction trap into the fingertip tube which was then sealed with cotton wool. The experiment was repeated four times, and 0.025% Tween-80 solution was used as a control. The pods were placed in a light incubator (Jiangnan Instrument, model: RXZ-380A) with a temperature of 25 ± 1 C, 70% relative humidity, and a 12 h light/dark photoperiod. The thrip death was observed, and the number of deaths recorded daily for 7 days. Thrips were considered dead if they were unable to stand upright and/or move forward when probed by a hair pencil tip. At the end of the experiment, dead adults were removed and placed in a petri dish with wet filter paper at the bottom for wet culture. Those that showed white mycelia grow around the thrips and typical conidiogenous structure observed by microscope were considered effectively infected.

### Inoculation of the Second-Instar Nnymphs

The leaf dipping method was adapted by cutting the leaves of common bean into small round blocks of about 1 cm^2^. The leaves of the common bean were soaked in the prepared spore suspension for 15 s and removed and dried naturally. Thirty second-instar nymphs were placed into a 25-mL conjoined plastic dip box (4.3 cm × 3.1 cm × 3 cm, with mesh glued to the holes at the top). The experiment was repeated four times, and 0.025% Tween-80 solution was used as the control. Nymphs were placed into a light incubator with 25 ± 1°C, relative humidity of 70%, and a 12 h light/dark cycle. Thrip death was observed, and the number of deaths was recorded daily for 7 days. At the end of the experiment, dead thrips were removed and placed in a petri dish with wet filter paper at the bottom for wet culture. Those that showed spore structure after incubation were considered effectively infected.

### Effects on *F. occidentalis* Fertility and Hatchability

Eggs of thrips are laid inside the bean pod tissue and are not easily visualized. Therefore, the number of eggs could not be determined. The difference in hatchability was determined by comparing the number of nymphs that appeared after the same number of thrips had laid their eggs over the same period of time. There were two experiments, the first involved treating the bean pods within which thrips had deposited eggs, and the other involved treating bean pods without eggs. The difference resulting from treatment after eggs were laid represented the difference in hatchability, while the difference resulting from the treatment before eggs were laid represented the difference caused by both fertility and hatchability.

In the first experiment, 20 female adult (2–3-day old) *F. occidentalis* were transferred by suction trap into the fingertip tube and sealed with cotton wool. After 24 h, the pods were placed into the spore suspension for 15 s and transferred into a new fingertip tube to identify the effects on the hatchability of eggs in pods. In the second experiment, pods were placed into the spore suspension for 15 s and transferred into a new fingertip tube. Twenty *F. occidentalis* adults female (2–3-day old) were transferred by suction trap into the fingertip tube, which was immediately sealed with cotton wool. Each experiment was repeated nine times, and 0.025% Tween-80 solution was used as the control. The pods were placed in a light incubator with a temperature of 25 ± 1°C, relative humidity of 70%, and 12 h light/dark cycle. The number of nymphs that emerged after 7-d was recorded.

### Statistical Analyses

*F. occidentalis* mortality was analyzed by one-way ANOVA using SPSS software (version 18.0; SPSS, Chicago, IL, United States). Tukey’s honestly significant difference test (*P* < 0.05) was used to analyze the survival rate of the adults and nymphs and the number of nymphs that hatched. Inverse sine square root transformation was used for the percentage data before the ANOVA analysis. All data are presented as mean ± SE. The corrected percent mortality was calculated by the Abbott’s formula (Abbott, 1925).

## Results

### Sequencing and Phylogenetic Analysis

**FIGURE 1:**
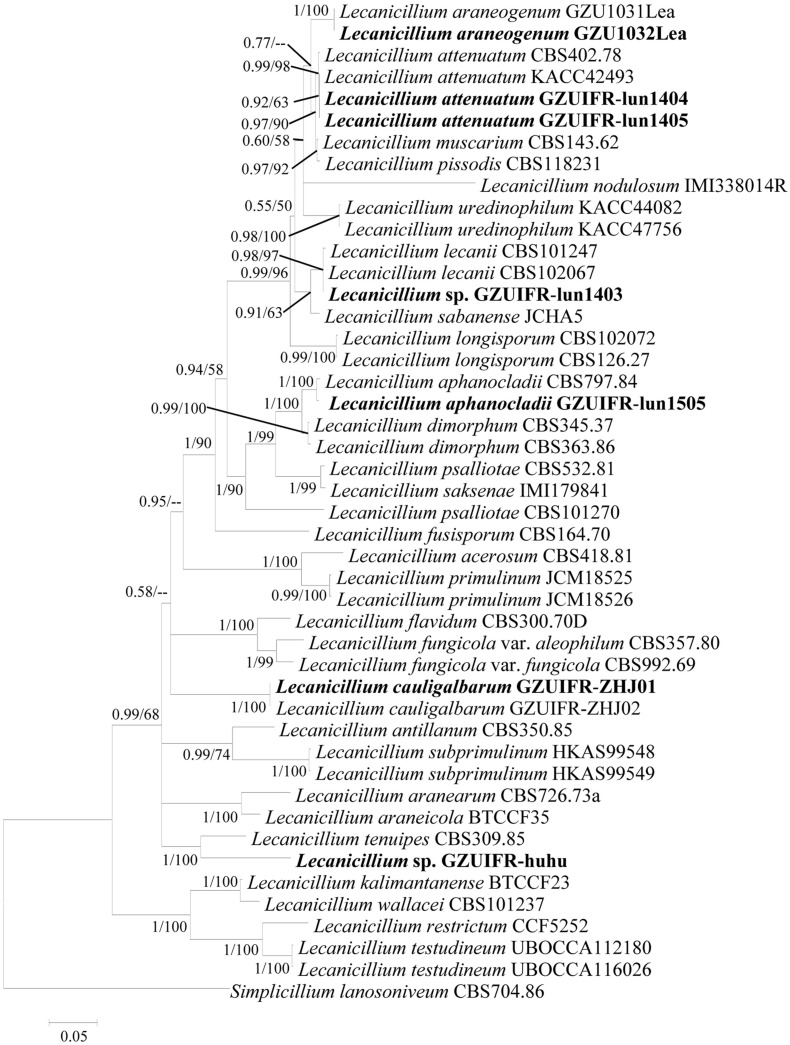
Phylogenetic analysis of isolated strains and related species based on partial ITS + *TEF* + *RPB1* + *RPB2* sequences. Statistical support values (≥0.5/50%) are shown at the nodes for Bayesian inference posterior probabilities/maximum-likelihood bootstrap support.

**FIGURE 2:**
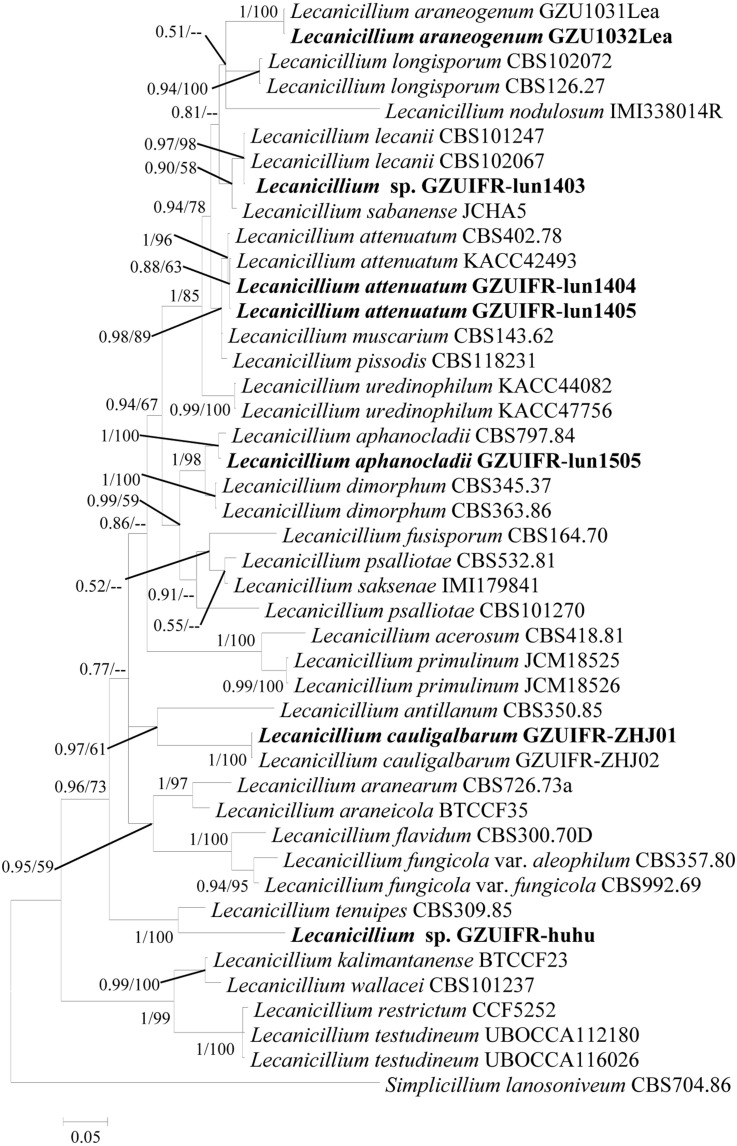
Phylogenetic analysis of isolated strains and related species derived from partial ITS + *RPB1* sequences. Statistical support values (≥0.5/50%) are shown at the nodes for Bayesian inference posterior probabilities/maximum-likelihood bootstrap support.

Two software programs (MRBAYES 3.2 and RAxML) were used to identify seven strains (Table 1) based on ITS, *SSU*, *LSU*, *TEF*, *RPB1*, and *RPB2* alone. Among these, ITS, *TEF*, *RPB1*, and *RPB2* were validated and useful for identifying the strains (phylogenetic incongruence with statistical measures of confidence and Supplementary Figures 1–4). The phylogenetic relationship analysis revealed that *LSU* and *SSU* could not be used for identifying the strains alone because of the small interspecific differences. The ITS + *TEF* + *RPB1* + *RPB2* partial sequence-based phylogenetic tree was formed using almost all *Lecanicillium* species (*L. evansii* was not in NCBI) and one *Simplicillium* species (*S. lanosoniveum*) as outgroup (Figure 1). The sequence dataset consisted of 2,735 bases, including inserted gaps (ITS: 523 bp; *TEF*: 772 bp; *RPB1*: 555 bp; and *RPB2*: 885 bp). *L. attenuatum* GZUIFR-lun1404 and *L. attenuatum* GZUIFR-lun1405 formed one branch in a polytomy together with a clade containing *L. attenuatum* (BI posterior probabilities 0.99, ML bootstrap 98%). *Lecanicillium* sp. GZUIFR- lun1403 formed one branch in a polytomy together with a clade containing *L. lecanii* (BI posterior probabilities 0.98, ML bootstrap 97%). *L. araneogenum* GZU1032Lea and *L. araneogenum*, *L. aphanocladii* GZUIFR-lun1505 and *L. aphanocladii*, and *L. cauligalbarum* GZUIFR-ZHJ01 and *L. cauligalbarum*, formed independent branches (BI posterior probabilities 1, ML bootstrap 100%). *Lecanicillium* sp. GZUIFR-huhu formed one branch in a polytomy together with a clade containing *L. tenuipes* (BI posterior probabilities 1, ML bootstrap 100%) (Figure 1). The other phylogenetic trees exhibited almost the same topology (Supplementary Figures 5–9 and Figure 2). No significant differences in topology were observed between BI and ML phylogenies.

### Virulence Comparison of the Seven Fungal Strains Infecting *F. occidentalis*

**FIGURE 3:**
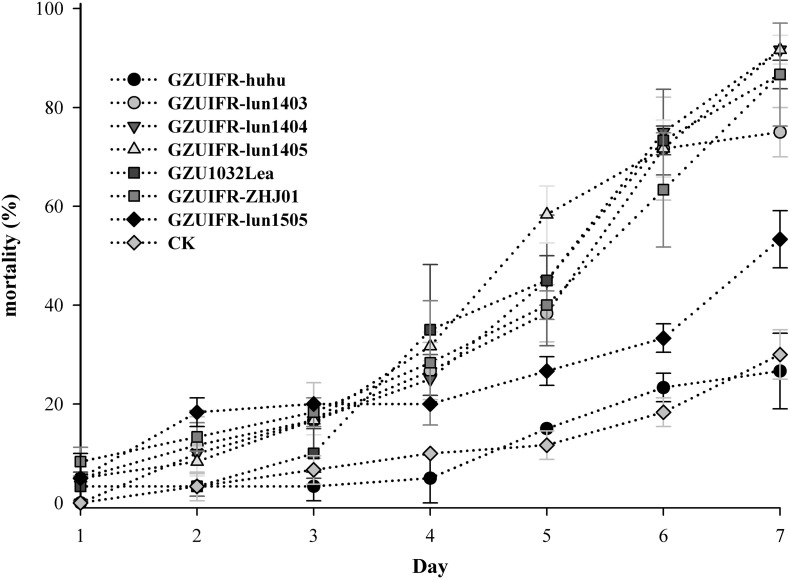
Mortality (%) of *F. occidentalis* adults after inoculation with different fungal species (mean ± SE).

**FIGURE 4:**
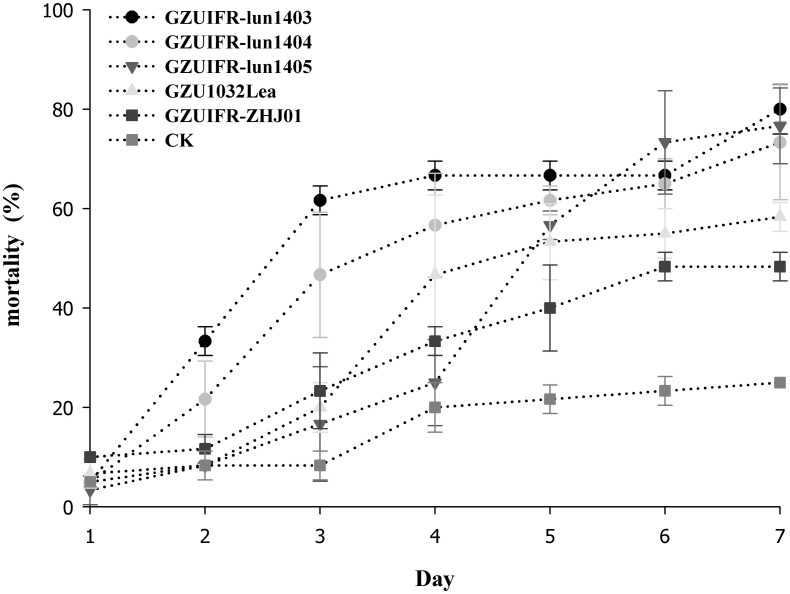
Mortality (%) of *F. occidentalis* second instar nymphs after inoculation with different fungal species (mean ± SE).

*Lecanicillium* sp. GZUIFR-huhu was non-virulent to *F. occidentalis*. The other six strains were pathogenic to *F. occidentalis*, but their virulence varied (Figure 3 and Supplementary Table 1). *L. attenuatum* strains GZUIFR-lun1404 and GZUIFR-lun1405 were the most virulent of the six strains from the five species. Approximately 8.33% of adult thrips survived after a 7-day exposure to these two *L. attenuatum* strains. Five strains showing effectiveness against *F. occidentalis* adult females were also pathogenic to second-instar nymphs. *L. attenuatum* strain GZUIFR-lun1405 was the most virulent, with approximately 23.33% of nymphs surviving after a 7-day exposure (Figure 4 and Supplementary Table 2).

**FIGURE 5:**
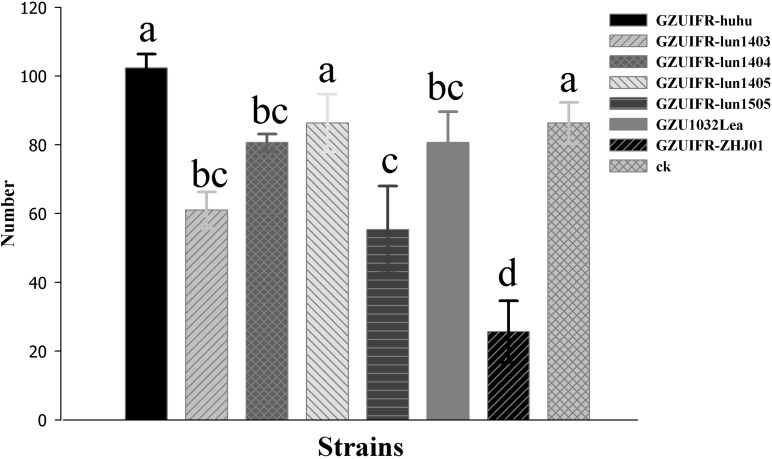
The number of nymphs produced by *F. occidentalis* adult females after the bioassay experiment. Bars represented by the same letter are not significantly different by Tukey–Kramer grouping for least squares means (*a* = 0.05) (*F*_7_,_23_ = 28.80; *p* = 0.0001).

**FIGURE 6:**
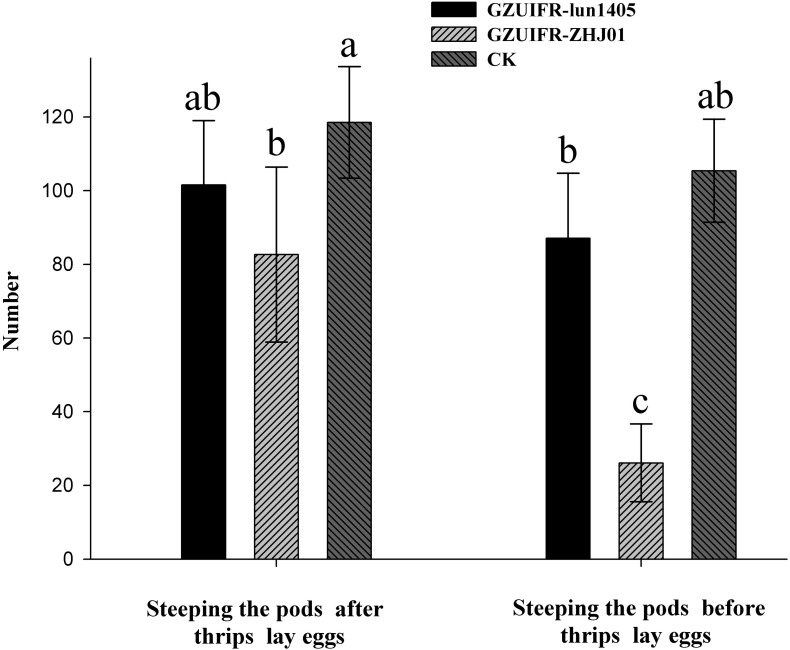
The numbers of *F. occidentalis* nymphs produced following different treatments. Bars represented by the same letter are not significantly different by Tukey–Kramer grouping for least squares means (*a* = 0.05) (*F*_5_,_52_ = 30.13; *p* = 0.0001).

Differences were found in the number of nymphs hatched by thrips after exposure to different strains. There were no significant differences among *Lecanicillium* sp. GZUIFR-huhu, *L. attenuatum* strain GZUIFR-lun1405, and control groups. The group treated with *L. cauligalbarum* strain GZUIFR-ZHJ01 had the fewest survivors, and their number of survivors was significantly fewer than those in other treatments and in the control group (*F*_7_,_23_ = 28.798; *p* < 0.0001) (Figure 5). *L. attenuatum* strain GZUIFR- lun1405 and *L. cauligalbarum* strain GZUIFR-ZHJ01 were selected for future analysis on the thrips fecundity. There was no significant difference among the number of nymphs hatched from experimental and control groups (*F*_2_,_26_ = 7.925; *p* = 0.0023) (Figure 6). However, the number of *F. occidentalis* nymphs significantly decreased when adult *F. occidentalis* were treated with *L. cauligalbarum* strain GZUIFR-ZHJ01 (*F*_2_,_26_ = 54.117; *p* < 0.0001) (Figure 6). These results show that the *L. cauligalbarum* strain GZUIFR-ZHJ01 affected fertility of the thrips.

## Discussion

DNA barcoding is proposed as an effective way to delineate and distinguish closely related species (Vu et al., 2019). A major advantage of DNA barcoding is that it usually does not require a trained technician and an experienced professional taxonomist. As part of Ophiocrdcipitaceae, *Lecanicillium* species are identified by ITS and multilocus (*TEF*, *RPB1*, *RPB2*, *LSU*, and *SSU*) sequence data. In general, combining barcodes shows clear benefits for species discrimination and provides higher discriminatory power than a single locus (Vu et al., 2016). However, we found no substantial benefit of using multilocus barcodes. In the present study, *LSU* and *SSU* could not successfully identify all species of *Lecanicillium* alone. Only *TEF*, *RPB1*, and *RPB2* were found to be useful for identifying all species alone. Our phylogenetic analyses reveal that an *RPB1*- based single locus tree showed better topology than did those based on *TEF* and *RPB2*. However, any one of these genes combined with ITS could also clearly distinguish the *Lecanicillium* genus. We believe a combination of ITS and *RPB1* is optimal for the efficient recognition of *Lecanicillium* spp. based on the richness of each sequence. Moreover, ITS and *RPB1* data already cover almost all species in the genus.

The two phylogenetic trees created in this study (Figures 1, 2) were formed with almost all *Lecanicillium* species. In previous reports, species aggregated with their corresponding sequences and maintained high approval ratings (Table 1). *Lecanicillium* sp. strain GZUIFR-lun1403 was identified as *L. lecanii* based on phylogenetic results. Although the *Lecanicillium* sp. strain GZUIFR-huhu was close to *L. tenuipes* in the phylogenetic tree, the frequency of base substitutions indicated a clear distinction from *L. tenuipes* (Figure 1). Therefore, we considered *Lecanicillium* sp. strain GZUIFR-huhu could be one new species. In all phylogenetic trees two *L. psalliotae* strains were not together in one branch. This indicated that the *L. psalliotae* strains require additional confirmation of the current taxon name by CBS.

Currently, the microbial agents used for *F. occidentalis* control are mainly *Beauveria bassiana* sensu lato (s.l.), *Metarhizium anisopliae* s.l., *Isaria fumosorosea*, and *Lecanicillium lecanii* strains (Wu et al., 2018). We found that *L. attenuatum*, *L. cauligalbarum*, *L. araneogenum*, and *L. aphanocladii* were pathogenic to *F. occidentalis*, but their virulence varied. This is the first report detailing the pathogenicity of these strains to *F. occidentalis*. The different host origin of our *Lecanicillium* strains do not appear to be directly associated with *F. occidentalis* virulence. Considering that *L. lecanii* has pathogenicity to many other thrips, we believe that the virulence of these strains to other thrips deserves further examination (Wu et al., 2018). In addition to direct pathogenicity, sublethal effects of the examined fungi on thrips, including fecundity, longevity, and feeding ability, may be the most important aspects of exposure to microbial control agents (Wu et al., 2018). Zhang et al. (2015) reported that a strain of *B. bassiana*, GZGY-1-3, significantly reduced the reproductive success of *F. occidentalis* and the subsequent fitness of their progeny. Additionally, one *M. anisopliae* strain significantly reduced the fecundity, egg fertility, and longevity of *Megalurothrips sjostedti* adults (Ekesi and Maniania, 2000). In this article, *L. cauligalbarum* strain GZUIFR-ZHJ01 did not produce the highest mortality rate in adults or nymphs, but significantly reduced the number of eggs laid by *F. occidentalis*. This effect could be an important factor to measure in the development of future *F. occidentalis* control projects. It is possible that the fungus competes with the host for nutrients and, consequentially, interferes with egg fertility at the physiological level. This sublethal effect could partially offset the relatively slow death rate of fungal infection compared to conventional insecticides and may play an important role in the control of thrips (Wu et al., 2018). The additional sublethal effects of the *L. cauligalbarum* strain GZUIFR-ZHJ01 on *F. occidentalis* need to be individually verified.

## Conclusion

Based on the performance of single barcodes and their combinations, we recommend combination of ITS and *RPB1* as the best barcodes for *Lecanicillium* identification. These two sequences individually do not currently cover all *Lecanicillium* species, so a combination of ITS and *RPB1* should be used for fast recognition of *Lecanicillium* spp. We have provided the first demonstration of the virulence of several *Lecanicillium* spp. against *F. occidentalis*, such as *L. attenuatum*, *L. cauligalbarum*, *L araneogenum*, and *L. aphanocladii*. In addition, the *L. cauligalbarum* strain GZUIFR-ZHJ01 caused both high *F. occidentalis* mortality and inhibited the fecundity of the pest.

## Data Availability Statement

The datasets presented in this study can be found in online repositories. The names of the repository/repositories and accession number(s) can be found in the article/Supplementary Material.

## Ethics Statement

*Frankliniella occidentalis* samples were maintained at our greenhouse at Institute of Entomology, Guizhou University, China – no permission was required.

## Author Contributions

YMZ performed the experiments, analyzed data, and wrote the article. XZ provided strains of fungus and revised the manuscript. JRZ designed the experiments and revised the manuscript. TJ analyzed the data. JQX performed the experiments. All the authors reviewed the manuscript.

## Conflict of Interest

The authors declare that the research was conducted in the absence of any commercial or financial relationships that could be construed as a potential conflict of interest.

## Footnotes

1 http://www.indexfungorum.org

2 https://www.ncbi.nlm.nih.gov/genbank/

3 https://treegraph.bioinfweb.info/

## Supplementary Material

Supplementary Figure 1 Phylogenetic analysis of the isolated strains and related species derived from partial ITS sequences. Statistical support values (≥0.5/50%) are shown at the nodes for Bayesian inference posterior probabilities/maximum-likelihood bootstrap support.

Supplementary Figure 2 Phylogenetic analysis of the isolated strains and related species derived from partial *TEF* sequences. Statistical support values (≥0.5/50%) are shown at the nodes for Bayesian inference posterior probabilities/maximum-likelihood bootstrap support.

Supplementary Figure 3 Phylogenetic analysis of the isolated strains and related species derived from partial *RPB1* sequences. Statistical support values (≥0.5/50%) are shown at the nodes for Bayesian inference posterior probabilities/maximum-likelihood bootstrap support.

Supplementary Figure 4 Phylogenetic analysis of the isolated strains and related species derived from partial *RPB2* sequences. Statistical support values (≥0.5/50%) are shown at the nodes for Bayesian inference posterior probabilities/maximum-likelihood bootstrap support.

Supplementary Figure 5 Phylogenetic analysis of the isolated strains and related species deriving from partial ITS + *TEF* sequences. Statistical support values (≥0.5/50%) are shown at the nodes for Bayesian inference posterior probabilities/maximum-likelihood bootstrap support.

Supplementary Figure 6 Phylogenetic analysis of the isolated strains and related species derived from partial ITS + *RPB2* sequences. Statistical support values (≥0.5/50%) are shown at the nodes for Bayesian inference posterior probabilities/maximum-likelihood bootstrap support.

Supplementary Figure 7 Phylogenetic analysis of the isolated strains and related species derived from partial ITS + *TEF* + *RPB1* sequences. Statistical support values (≥0.5/50%) are shown at the nodes for Bayesian inference posterior probabilities/maximum-likelihood bootstrap support.

Supplementary Figure 8 Phylogenetic analysis of the isolated strains and related species derived from partial ITS + *TEF* + *RPB2* sequences. Statistical support values (≥0.5/50%) are shown at the nodes for Bayesian inference posterior probabilities/maximum-likelihood bootstrap support.

Supplementary Figure 9 Phylogenetic analysis of the isolated strains and related species derived from partial ITS + *RPB1* + *RPB2* sequences. Statistical support values (≥0.5/50%) are shown at the nodes for Bayesian inference posterior probabilities/maximum-likelihood bootstrap support.

Supplementary Table 1 Comparison of the influences of test strains on the mortality rate (%) of *F. occidentalis* adults.

Supplementary Table 2 Comparison of the influences of test strains on the mortality rate (%) of *F. occidentalis* second instar nymphs.

## Abbreviations

Spp.: species pluralis
GZAC: the Institute of Fungal Resources of Guizhou University
rDNA: ribosomal DNA
LSU: the large subunits of the rDNA
SSU: the small subunits of the rDNA
TEF: the transcription elongation factor-1 α
ITS, ITS1–5.8S rDNA–ITS2 region: the first and the internal transcribed spacers
RPB1: DNA-directed RNA polymerase II subunit rpb1
RPB2: DNA-directed RNA polymerase II subunit rpb2.
