# Supplementary material for: Fast Recognition of Lecanicillium spp., and Its Virulence Against Frankliniella occidentalis
Source: Front Microbiol. 2020 Oct 22;11:561381. doi: 10.3389/fmicb.2020.561381 (PMC7642397; doi:10.3389/fmicb.2020.561381)
Supplement: Supplementary Figure 1 — Phylogenetic analysis of the isolated strains and related species derived from partial ITS sequences. Statistical support values (≥0.5/50%) are shown at the nodes for Bayesian inference posterior probabilities/maximum-likelihood bootstrap support. [file Data_Sheet_1.docx]

**Additional Table 1.** Comparison of the influences of test strains on the mortality rate (%) of *F. occidentalis* adults

| Strain | Mortality | | | |
| --- | --- | --- | --- | --- |
|  | 1 d* | 3 d** | 5 d*** | 7 d**** |
| GZUIFR-huhu | 0 a | 3.33±1.67 c | 15±0 de | 26.67±4.41 d |
| GZUIFR-lun1403 | 5±2.89 a | 16.67±4.41 ab | 38.33±3.33 bc | 75±2.89 b |
| GZUIFR-lun1404 | 0 a | 16.67±1.67 ab | 45±7.64 ab | 91.67±1.67 a |
| GZUIFR-lun1405 | 5±2.89 a | 16.67±1.67 ab | 58.33±3.33 a | 91.67±1.67 a |
| GZU1032Lea | 3.33±1.67 a | 10.00±2.89 abc | 45±2.89 ab | 86.67±1.67ab |
| GZUIFR-ZHJ01 | 8.33±1.67 a | 18.33±1.67 | 46.67±1.67 ab | 86.67±6.01ab |
| GZUIFR-lun1505 | 5±2.89 a | 20.00±0 a | 26.67±1.67 cd | 53.33±3.33 c |
| CK | 0 a | 6.67±1.67 bc | 11.67±1.67 e | 30%±2.89 d |

The data in the table are the means±*SEs*, and the numbers in the same column with different letters are significantly different (Tukey’s test, *P*<0.05). * (*F*_7, 23_=2.84; *p*=0.0395). ** (*F*_7, 23_=6.82; *p*=0.0007). *** (*F*_7, 23_=26.10; *p*=0.0001). ****; (*F*_7, 23_=47.29; *p*=0.0001).

**Additional Table 2.** Comparison of the influences of test strains on the mortality rate (%) of *F. occidentalis* second instar nymphs

| Strain | Mortality | | | |
| --- | --- | --- | --- | --- |
|  | 1 d* | 3 d** | 5 d*** | 7 d**** |
| GZUIFR-lun1403 | 5±0 a | 61.67±1.67 a | 66.67±1.67 a | 80±2.89 a |
| GZUIFR-lun1404 | 5±0 ab | 46.67±7.26 ab | 61.67±1.67 a | 73.33±6.67 ab |
| GZUIFR-lun1405 | 3.33±2.89 b | 16.67±6.67 c | 56.67±1.67 ab | 76.67±4.41 a |
| GZU1032Lea | 6.67±2.89 ab | 20±2.89 c | 53.33±4.41 ab | 58.33±1.67 bc |
| GZUIFR-ZHJ01 | 10±0 a | 23.33±4.41 bc | 45±5.00 b | 48.33±1.67 c |
| CK | 5±0 ab | 8.33±1.67 c | 21.67±1.67 c | 25±0 d |

The data in the table are the means±*SEs*, and the numbers in the same column with different letters are significantly different (Tukey’s test, *P*<0.05). * (*F*_5, 17_=2.78; *p*=0.0682). ** (*F*_5, 17_=17.26; *p*=0.0001). *** (*F*_5, 17_=29.16; *p*=0.0001). ****; (*F*_5, 17_=29.43; *p*=0.0001).

**Additional Figure 1.** Phylogenetic analysis of the isolated strains and related species derived from partial ITS sequences. Statistical support values (≥ 0.5/50%) are shown at the nodes for Bayesian inference posterior probabilities/maximum-likelihood bootstrap support.

**Additional Figure 2.** Phylogenetic analysis of the isolated strains and related species derived from partial *TEF* sequences. Statistical support values (≥ 0.5/50%) are shown at the nodes for Bayesian inference posterior probabilities/maximum-likelihood boostrap support.

**Additional Figure 3.** Phylogenetic analysis of the isolated strains and related species derived from partial *RPB1* sequences. Statistical support values (≥ 0.5/50%) are shown at the nodes for Bayesian inference posterior probabilities/maximum-likelihood boostrap support.

**Additional Figure 4.** Phylogenetic analysis of the isolated strains and related species derived from partial *RPB2* sequences. Statistical support values (≥ 0.5/50%) are shown at the nodes for Bayesian inference posterior probabilities/maximum-likelihood boostrap support.

**Additional Figure 5.** Phylogenetic analysis of the isolated strains and related species deriving from partial ITS *+TEF* sequences. Statistical support values (≥ 0.5/50%) are shown at the nodes for Bayesian inference posterior probabilities/maximum-likelihood boostrap support.

**Additional Figure 6.** Phylogenetic analysis of the isolated strains and related species derived from partial ITS+*RPB2* sequences. Statistical support values (≥ 0.5/50%) are shown at the nodes for Bayesian inference posterior probabilities/maximum-likelihood boostrap support.

**Additional Figure 7.** Phylogenetic analysis of the isolated strains and related species derived from partial ITS+*TEF*+*RPB1* sequences. Statistical support values (≥ 0.5/50%) are shown at the nodes for Bayesian inference posterior probabilities/maximum-likelihood boostrap support.

**Additional Figure 8.** Phylogenetic analysis of the isolated strains and related species derived from partial ITS+*TEF*+*RPB2* sequences. Statistical support values (≥ 0.5/50%) are shown at the nodes for Bayesian inference posterior probabilities/maximum-likelihood boostrap support.

**Additional Figure 9.** Phylogenetic analysis of the isolated strains and related species derived from partial ITS+*RPB1*+*RPB2* sequences. Statistical support values (≥ 0.5/50%) are shown at the nodes for Bayesian inference posterior probabilities/maximum-likelihood boostrap support.
